# SPOUSE OR CAREGIVER? EXAMINING CORRELATES AND CONSEQUENCES OF SPOUSES BEING IDENTIFIED AS CANCER CAREGIVERS

**DOI:** 10.1093/geroni/igz038.1051

**Published:** 2019-11-08

**Authors:** Kristin Litzelman

**Affiliations:** University of Wisconsin-Madison, Madison, Wisconsin, United States

## Abstract

Spouses provide critical support to cancer survivors, but are not always identified as caregivers. This study sought to understand the differences between such spouses. Using the Medical Expenditures Panel Survey 2016 Experiences with Cancer Supplement, spousal dyads in which one spouse had cancer were identified (n=670). Cancer survivors reported which family members or friends, if any, provided care during or after their cancer treatment. Survivor and spouse sociodemographic characteristics and spouse psychosocial characteristics including depression (Patient Health Questionnaire-2), distress (Kessler-6), and self-rated health were self-reported in the survey. The proportion of spouses identified as caregivers was calculated and compared with those not identified as caregivers on dyadic characteristics. Multivariable logistic regressions compared spouses’ depression, distress, and self-rated health by identification as a caregiver. All analyses employed survey weighting. Most married cancer survivors reported that their spouse was a caregiver (32%) or that they did not have a caregiver (65%); very few did not list their spouse as a caregiver (3%). Survivors who were white (non-Hispanic) or off treatment were less likely to report that their spouse was a caregiver (30% vs. 46%, p0.10; 9.6% vs 11.1%, p>0.10; and 46% vs 50%, p>0.10, respectively). The findings suggest that spouses’ experiences may be similar regardless of whether they are considered a caregiver, with implications for research and service delivery.

